# Prospective evaluating the appropriate use of piperacillin /tazobactam in cardiac center of a tertiary care hospital

**DOI:** 10.1186/s13019-020-01109-y

**Published:** 2020-05-01

**Authors:** Sanaa Saeed Mekdad, Leenah AlSayed

**Affiliations:** 1 Department of Clinical Pharmacy, King Fahad Medical City, PO Box 59046, Riyadh, 11525 Saudi Arabia; 2 Department of In-patient Pharmacy, King Fahad Medical City, Riyadh, 11525 Saudi Arabia

**Keywords:** Drug utilization review, Piperacillin-Tazobactam, Rational use of pip/Taz, Antibiotic stewardship program, De-escalation of antibiotics

## Abstract

**Background:**

The appropriate use of Piperacillin/Tazobactam (Pip/Taz), including the correct dose, escalating and or de-escalation according to the microbiological culture is essential to reduce the antibiotic resistance. Resistant to antimicrobials in a major global problem and contributes significantly to morbidity, mortality and cost of care. Guidelines exists to ensure appropriate use of Pip/Taz. Antibiotics Stewardship guidelines (https://apps.who.int/iris/bitstream/handle/10665/329404/9789241515481-eng.pdf) provides a detailed recommendation with regards to initiation, monitoring and escalation and de-escalation based on final culture results. Appling such guidelines ensures a more proper utilization of the empiric uses of antibiotics used in the hospital-based setting. Use of Pip/Taz in cases of suspected infection postoperatively is common practice in the cardiac surgery ward where this study was conducted.

**Methods:**

This was a prospective cohort study involving all patients who were admitted to the cardiac surgery unit of a tertiary care center. All patient prescribed at least 1 day of Pip/Taz as an empirical therapy were included and prospectively observed. We aimed to evaluate the use of Pip/Taz and its appropriateness based on Antibiotics Stewardship guidelines (ASG). Any deviation from the guidelines in initiation, escalation, de-escalation based on culture and sensitivity results was considered inappropriate use. Four patients died (1.3%) early as result of complications for surgery but included in the analysis. The study was conducted from October 2017 to October 2018.

**Results:**

Of the 300 patients who received Pip/Taz Cultures were done in 250 patients (83%). The overall appropriate use of Pip/Taz was seen in 166 patients (55.3%).

**Conclusion:**

The empirical use of Pip/Taz in the surgical cardiac unit was largely inappropriate and not entirely driven by the culture test results. Interventions are needed to optimize the use of Pip/Taz including appropriate culture and sensitivity driven use and timely de-escalation or de-escalation when indicated. This will prevent emergence of resistance and reduce the patient toxicity and financial costs.

## Introduction

The broad-spectrum antibiotics, such as Pip/Taz, play an essential role in the empirical therapy of serious infections [1].

Pip /taz is a combination of β-lactam/β-lactamase inhibitor and has a broad spectrum of antibacterial activity including most Gram-positive and Gram-negative aerobic bacteria and anaerobic bacteria and is effective for many polymicrobial infections [1].

Pip/Taz is useful against a variety of respiratory infection, intra-abdominal, skin and soft tissue infections, febrile neutropenia, and bloodstream infections (BSI) which are among the most frequent complications in neutropenic cancer patients caused by Gram-negative rods which associated with high mortality [2].

Improper use of these antibiotics is common and costly, as physicians often use broad-spectrum antibiotics when a narrower-spectrum agent would work. Overuse of broad-spectrum agents also has an important role in growing worldwide bacterial resistance which has become a serious problem and adds to the overall costs of medical care with increased side effects [3].

.

The Infectious Diseases Society of America (IDSA) has published stewardship guidelines to optimize antibiotic use, using cost-effective interventions, to minimize antibiotic resistance, minimize side effects and control *Clostridium difficile* infections [4–6].

Various strategies have offered for antimicrobial stewardship programs. Like staff education, formulary restrictions and substitutions, early de-escalating, early converting parenteral to oral, and practice guidelines that consider local microbiology and resistance [7].

One useful strategy is to decrease inappropriate use of broad spectrum antibiotics by restricting its use to infectious diseases consultant to get approval for dispensing [8–10].

By identifying the inappropriate use of pip /taz, further regulations can be implemented to optimize the use of this medication and thus decreasing the antimicrobial drug resistance which contribute to better patient outcomes. In addition, if appropriate use of Pip/Taz is established, unnecessary hospital costs can be reduced. Hence, we conducted this study to estimate the appropriate use of Pip/Taz utilization in the surgical cardiac unit using Antibiotic stewardship guidelines as a reference.

## Methods

We included all patients who were prescribed Pip/Taz for more than 24 h in cardiac surgery unit of a tertiary hospital. The patient’s demographic data, microbiology tests, renal function tests and the dose adjustment were all recorded from patients’ electronic medical records.

We prospectively followed their clinical course after the empiric initiation. Final microbial culture and sensitivity and escalation or de-escalation/discontinuation based on results and (ASG). The overall appropriate use was measured.

Analysis of the appropriateness of Pip/Taz indication, dose, frequency and duration of therapy was based on the available ASG by the Infectious Diseases Society of America.

The guidelines summary is detailed below.

Pip/Taz prescription was deemed appropriate if:

1. Pip/ Taz was selected as an empirical therapy according to the hospital’s guidelines
2. It was used as an alternative antibiotic with a narrow spectrum after culture result.
3. The dose was adjusted with renal function of the patient.
4. Pip/ Taz was discontinued once the culture was negative or showed a resistant organism.
5. If one of these conditions was not met, the prescription was considered inappropriate.

### Inclusion criteria

All adult patients 18 years or older who were admitted to cardiac surgery ward from October 2017 to February 2019 and had received Pip/Taz for 1 day or more were included in the study. An informed consent was obtained.

### Exclusion criteria

Patients who had received Pip/Taz for less than 1 day.

Patients less than 18 years old or who refused to give consent.

### Data analysis

The analysis was performed using SPSS software version 21. Analysis was done by using simple descriptive statistics using mean and median for continuous data.

The institutional review board at KFMC approved the study.

## Results

A total of 300 patients who received at least one of day of Pip/Taz were included. Baseline characteristics, and indication of the received Pip/Taz are illustrated in Table 1.

**Table 1:** Baseline characteristics (*N* = 147)

Characteristic	N (%)
Male	210 (71.4%)
Female	84 (26.6%)
Mean age	62.9+/_11.3
Creatinine clearance	
● More than 40 ml/min	220 (75%)
● Less than 40 ml/min	60 (20%)
RRT	14 (5%)
Mean duration of therapy	8.1+/_4.7 days

*RRT* Renal Replacement Therapy

Four patients died shortly post-surgery, their initial empiric therapy was considered appropriate and were included in the study. The main reason for the initiation of Pip/Taz was empirical therapy. Only 14 cases (6.7%) were prescribed PipTaz according to culture and sensitivity tests from the beginning. Two hundred eighty-six patients (95.3%) of the cases were prescribed empirically for one spike of fever, Chest x-ray suggestive of post-operative pneumonia and or high white blood cell count (WBC). With regards to empiric use thirty six (12%) was considered inappropriate as patients continued these antibiotics for more than 5 days without any microbial test or ID (Infectious disease) consultation. In 250 patients (83.3%) culture was sent and empiric use was deemed appropriate. Subsequent follow up showed that Pip/tazo use was appropriate in 166 (55.30%).

**Fig. 1:**
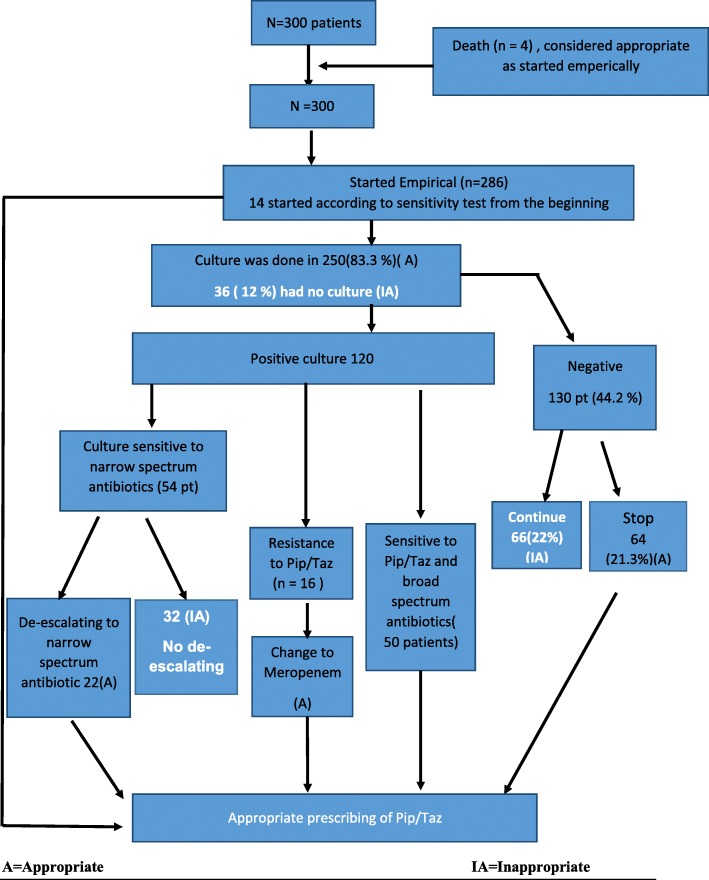
Consort diagram: The overall flow of patients

After the culture-test results, 130 (43.3%) had no microbial growth on any culture. In 64 (21.3%) pip/taz was discontinued based on the results and hence deemed appropriate. While 66 (22%) patient continued the Pip/Taz despite negative cultures and deemed inappropriate (Fig. 1).

One hundred twenty patients had a positive microbial culture, de-escalation to narrow spectrum antibiotics was indicated in 54 (18%) patients and was done on time (within 24 h) in 22 pts. (7.3%) but delayed > 24 h after the availability of culture-sensitivity test results was continued in 32 (10.6%) patients and deemed inappropriate.

Sixteen cases (5.3%) were resistant to Pip/Taz according to sensitivity test and Pip/Taz was changed to Meropenem and were considered appropriate. 50 (16.7%) cases were sensitive to Pip /Taz and other broad-spectrum antibiotics and continuation of treatment was justified.

Fourteen patients (4.7%) required dose adjustment based on renal functions. Most inappropriateness in dosing (8 cases) was mainly due to low dose in renal replacement therapy (5 cases) as about 25–30% of the drug is removed by dialysis and there is a need to add 0.75 mg post dialysis three times /week, while three patients had renal impairment and needed to adjust the dose with renal clearance. In 6 patients with renal issues the dosing remained appropriate.

In summary Antibiotics use was considered inappropriate if they are prescribed empirically without a culture-test request, dose was not adjusted with renal function o renal replacement therapy, if their use continued in the presence of a negative culture-test result in cases of delayed de-escalation (Fig. 1) (Tables 2 and 3).

**Table 2:** Culture and Infectious consultation

Criteria	Work done	Work not done
Microbiology and culture test within 24 h	250 (83.3%)	50 (16.7%)
Infectious disease consultation	178 (59.3%)	122 (40.7%)
Starting as an empiric therapy in accordance with ADSA guidelines	286 (95.3%)	14 (4.7%)
Dosing, dose adjustments accordance to a drug data base reference (Lexi comp).	14(5%)	8 (2.7%)

**Table 3:** Summary of Piperacillin/Tazobactam appropriate usage

Criteria	Appropriate n (%)	Inappropriate
Starting according to microbiology test	14 (4.7%)	
Continued according to the microbiological test	66\130 (22%)	
Discontinuation of therapy after negative culture	64/130 (21.3%)	
De-Escalation to an alternative after culture data showed sensitivity to narrow spectrum antibiotic	22/54 (7.3%)	
Discontinuation once the culture data showed *Pip/Taz* resistant organism	16 (5.3%)	
Patients had appropriate Pip/Taz use	166 (55.3%)	134 (44.7%)

## Discussion

Pip/Taz plays an important role as empiric therapy for serious infections post-surgery. Available clinical trials have marked the safety and efficacy of Pip/Taz monotherapy in the treatment of moderate to severe infections including bacteremia associated with post-operative nosocomial infections, intravenous line associated, nosocomial respiratory tract infections, febrile neutropenia, intra-abdominal and peritoneal infections, and complicated wound infections [11, 12].

We attempted to prospectively study the journey the postoperative cardiac patient who is started on empiric Pip/taz and address the appropriateness of use of Pip/Taz in these patient and potential areas of improvement.

The empiric use of pip/taz as monotherapy was common in postoperative patients demonstrating signs of possible infection. The main reasons for initiating Pip/Taz was use was fever, or high level of white blood cells (WBCs). However potential for inappropriate use exists in complicated post-operative cardiac cases.

We were able to demonstrate the potential inappropriate use is this setting is a genuine concern.

Overall appropriate use was demonstrated in 55% of cases.

The common causes include inadequate initial work up, failure to adjust dosing and inappropriate de-escalation of antibiotics after the microbial culture results. At the time of initiation 12% of cases Pip/Taz use was inappropriate as the antibiotic was started without a culture and sensitivity test.

In a review of the literature. A study by Thomas 2015 addressed the duration of empiric antibiotic therapy in adult ICUs, 333 of 660 (50%) of the empiric antibiotics were continued for at least 72 h and half of all empiric antibiotics ordered in critically ill patients are continued for at least 72 h in absence of adjudicated infection [13].

A study by Rattanaumpawan and Youssif concluded that Piperacillin/tazobactam, Imipenem, and Meropenem were inappropriately used in 50% of hospitalized patients similar to our study [14, 15].

Two studies by Khan and Wiskirchen the rate of appropriate prescription of Pip/Taz as empiric therapy was 57.0% Most inappropriate prescriptions were found mostly in surgical wards and the surgical intensive care units which seems similar to the setting of our patients [16, 17].

Another study by Youssif found that the continuation of antibiotics based on culture and sensitivity results was justified in only 22 patients out of 163 and only 33/64 patient the antibiotics was de-escalated when indicated by culture and sensitivity results. They concluded that the use of broad-spectrum antibiotics in surgical floors at a tertiary care hospital was largely unjustified by culture-test result [15].

The rate of Pip/Taz appropriate use post-cultures was 62.4%. The inappropriateness after the culture and sensitivity results was mainly due to the inability to de-escalate therapy in many cases, and the continuation of Pip/Taz therapy despite negative cultures.

As compared to the other studies, our study showed a relatively high percentage of negative cultures post culture work-up. Most patients enrolled in other studies were elderly while mean age in our study was 62.9+/− 11.3. Nonspecific sepsis like symptoms, renal impairment, metabolic acidosis, and dehydration are toxic effects of pip/Taz common in the elderly population. Failure to discontinue antibiotics post negative cultures expose this group of patients to unnecessary potential toxicity [16].

Special care is needed in patients with renal impairment or those requiring renal replacement therapies. Although not a major cause of inappropriate use in our study. Other studies have show this to be n important cause. With regards of appropriate dosing of Pip/Taz a study involving 185 patients the rate of appropriateness of dosing in renal impaired patients was 77.8%.

Finally, we noticed about 15% patients were prescribed Pip/Taz without a culture, sensitivity test request. We found that the improper empirical prescribe of Pip/Taz without culture work up is a common practice among cardiac surgeons with risk of infection after surgery, or even in patients with signs and symptoms of sepsis like fever and elevated white-blood-cell count.

The utilization and appropriate use of empiric antibiotics can be improved. Application of guidelines have been associated with better overall appropriate use, antibiotics Stewardship guidelines application has shown to improve overall correct use of antibiotics and potentially the overall cost [18].

In general, the physicians require good knowledge and experience about the use of antibiotics for management of nosocomial infections as per available guidelines. Differential diagnosis and the comorbidities of the critical ill subjects involved should be considered. It is important to modify antimicrobial therapy according to the sensitivity results in order to reduce costs, decrease the development of resistance and minimize the incidence of side effects and shortage [17, 19–21].

Physicians should be vigilant about the need to stop and deescalate therapy when appropriate and early consultations with infectious disease services should be encouraged when nosocomial infections are suspected or documented. Modifications or de-escalation of antimicrobials help in minimizing the cost and reduce resistance.

## Conclusion

Our study highlights that there is a major potential for improvement in the appropriate utilization of use of Pip/Taz specially in surgical wards. This improvement can be a multilevel starting with appropriate initiation of empiric therapy to observing the culture and sensitivity results, appropriate escalations and de-escalations according to results. Early discontinuations when appropriate and being vigilant for dose modifications as many patients can have renal impairment post operatively. These multistep interventions will result is overall appropriate utilization of these antibiotics. It will certainly reduce the incidence of overall resistance, potentially reduce toxicity and can reduce the overall health care costs. Also the application of guidelines like implementation of the antimicrobial stewardship program will help in improving the clinical practice. Education and training the staff aiming at a more appropriate use of antibiotics use has been identified [22, 23].

## Data Availability

Not applicable.

## Abbreviations

IDSA: Infectious Disease Society of America
IRB: The Institutional Review Board
WBCs: White Blood Cells
